# SNHG3/hsa-miR-455-5p Axis-mediated High Expression of MTHFD2 Correlates with Tumor Immune Infiltration and Endometrial Carcinoma Progression

**DOI:** 10.7150/ijms.81962

**Published:** 2023-07-03

**Authors:** Sa Wu, Weisong Cai, Yanli Li, Wenfu Tan, Yichong Yuan, Zhigang Zhou, Jie Shi, Xiaotian Liu, Han Gao

**Affiliations:** 1 Department of Gynaecology Ⅱ, Maternal and Child Health Hospital of Hubei Province, Tongji Medical College, Huazhong University of Science and Technology, Wuhan, China; 2 Department of Otorhinolaryngology, Head and Neck Surgery, Zhongnan Hospital of Wuhan University, Wuhan, China

**Keywords:** MTHFD2, endometrial carcinoma, prognosis, biomarker, lncRNAs

## Abstract

**Purpose**: Endometrial carcinoma (EC) is one of the three most common female genital tract cancers, and it contributes to the leading deaths of gynecologic cancer. MTHFD2 was reported up-regulated and associated with poor prognosis in many malignancies. However, its biological functions and mechanisms in EC are unclear. The present study aimed to identify the biological functions and potential molecular mechanisms of MTHFD2 in EC.

**Methods**: The gene expression and information of patients used in this study were derived from TCGA, GEO and HPA databases. KM survival analysis was used to explore the clinical outcomes of EC patients and correlation analysis was applied to find the correction between MTHFD2 expression level and immune infiltration in EC. We used GO and GSEA analysis to explore the biological functions and mechanisms of MTHFD2. The CCK8 assay, the colony formation assay and the transwell migration assay were conducted to validate the function of MTHFD2 in EC cells. We applied STRING to find the protein that interacted with MTHFD2. Finally, ENCORI was used to explore the potential upstream regulation of MTHFD2 in EC and it was validated in EC cells.

**Results**: In the present study, we found that MTHFD2 was up-regulated in EC and its high expression level was associated with patients' poor prognosis and adverse clinical parameters. MTHFD2 level was shown to be correlated with immune infiltration. Knockdown of MTHFD2 inhibited the malignant phenotype of HEC-1A and Ishikawa cells, including proliferation, colony formation and migration. Furthermore, we found the SNHG3/hsa-miR-455-5p axis as the potential upstream of MTHFD2.

**Conclusion**: SNHG3/hsa-miR-455-5p axis-mediated high expression of MTHFD2, and the MTHFD2 expression level was associated with tumor immune infiltration and endometrial carcinoma progression. Knockdown of MTHFD2 significantly inhibited the malignant phenotype of EC cells. MTHFD2 may be a valuable predictive biomarker, and targeting MTHFD2 may be an effective way to improve the therapeutic effect in EC.

## Introduction

Endometrial carcinoma (EC), also called uterine corpus endometrial carcinoma (UCEC), is one of the three most common female genital tract cancers, which is derived from the Endometrial epithelium. There could be 319,500 new cases per year worldwide, with a mortality rate of more than 23%^1^. The treatment for EC includes surgery, radiotherapy, chemotherapy, hormone therapy and other auxiliary means for comprehensive treatment^2^. However, the high local recurrence rate, high metastasis rate and hormone resistance are still the dilemmas of clinical treatment. What's more, conservative treatment to preserve fertility is very important for young EC patients at an early-stage EC^2^. Therefore, it is urgent to explore the potential molecular mechanisms of the occurrence and find novel biomarkers and effective therapeutic targets.

Methylenetetrahydrofolate dehydrogenase 2 (MTHFD2) is one of the key enzymes of folate one-carbon metabolism. Products of its catalytic process, including S-adenosylmethionine production, cellular nucleotide and amino acid (serine and glycine), are vital for supporting the malignant growth of cancer cells^3^. Recent studies have revealed its nonenzymatic effect in the aspect of DNA repair, RNA translation and redox homeostasis^4^-^7^. Several studies have reported that MTHFD2 was significantly up-regulated in many malignancies and its high expression was related to a poor prognosis^8^-^11^. However, the expression level of MTHFD2 in EC and its role in the progression of EC remain unclear. Therefore, it's valuable to study the effect of MTHFD2 and the relative mechanism in EC.

**Figure 1:**
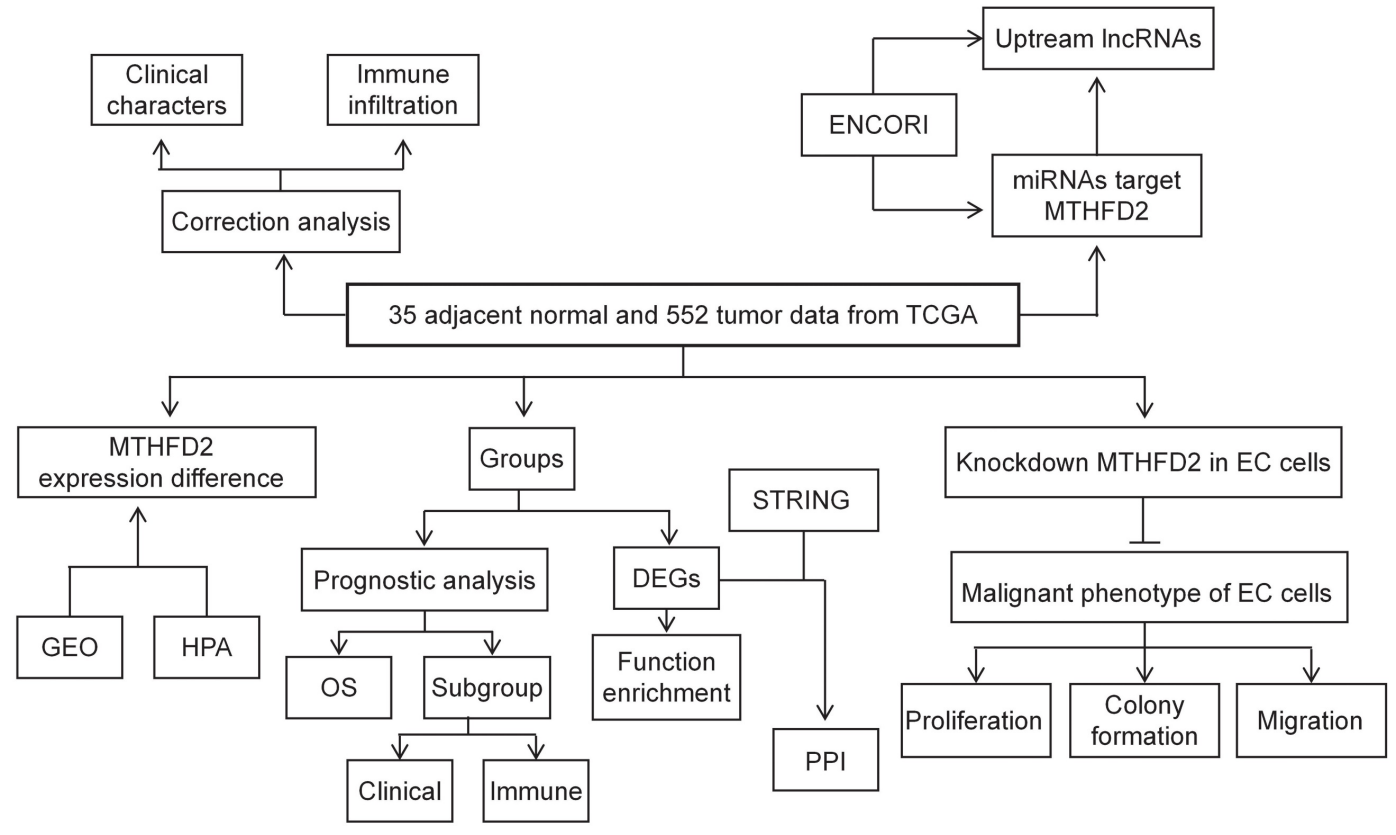
The flow process diagram.

In the present study, TCGA, GEO, HPA, UALCAN datasets, and Kaplan-Meier (KM) plotter web were applied to analyze the MTHFD2 expression level and its correlation with patients' prognosis and clinical significance. In addition, the association between MTHFD2 expression level and immune infiltration was tested to study the potential mechanisms involved in MTHFD2 regulation in the progression of EC. To further explore the function of MTHFD2 in EC, we established the MTHFD2 knockdown endometrial carcinoma cells and conducted the experiments in *vitro*. Through correlation, expression, and survival analysis, noncoding RNAs (ncRNAs) were screened to result in the up-regulation of MTHFD2 in EC. Finally, the SNHG3/hsa-miR-455-5p axis was founded as the most possible upstream ncRNA-related pathway of MTHFD2 in EC (Fig. 1). To conclude, this study indicated the potential role of MTHFD2 in EC and provided new clues and targets for EC diagnosis and treatment.

## Methods

### Gene expression, correlation and survival analysis

Gene expression profiles of EC containing 35 adjacent normal tissues and 552 tumor tissues were downloaded from the TCGA database. Gene expression profiles of EC containing 33 adjacent normal tissues and 64 tumor tissues were derived from the GEO database (GSE106191). R software (v4.2.1.) was used for expression analysis, “survminer”, “ggplot2”, and “survival” R packages were used for KM survival curves. We applied Log-rank analysis to assess the significance, and univariate cox proportional hazards regression to test the p-values and hazard ratio (HR) in KM curves. The R package “ggstatsplot” was used for two-gene correlation analysis, and Pearson's or Spearman's correlation analysis was used to estimate quantitative variables correlation. Finally, the MTHFD2 gene expression level was validated in external databases, the Gene Expression Omnibus database (GEO). UALCAN Database (http://ualcan.path.uab.edu/)^12^ was applied to assess the MTHFD2 gene expression level of EC in TCGA database.

### Kaplan-Meier Plotter Database Analysis

The online database KM plotter^13^ (http://kmplot.com/analysis/) was used to study the prognostic roles of miRNAs in EC.

### PrognoScan Database Analysis

The association between MTHFD2 expression and overall survival (OS) in EC was tested by the PrognoScan database (http://www.abren.net/PrognoScan/)^14^.

### The Human Protein Atlas Database Analysis

The human protein atlas (HPA) (https://proteinatlas.org/) is an online website containing protein expression profile information of human normal tissue and tumor tissue^15^. In this finding, the protein expression of MTHFD2 in EC was investigated by HPA. Each image was respectively scored by two pathologists using Image J(v1.8.0) with the following rules: the scores= staining intensity* the proportion of positive cells(staining intensity:0, negative; 1, low positive; 2, positive; 3, high positive; the percentage contributionof positive cells:1, < 10%; 2, 10-35%; 3, 35-70%, 4, > 70%). Finally, we normalized the score.

### ENCORI Analysis

The Encyclopedia of RNA Interactomes (ENCORI) database was used to explore the correlation between miRNA-mRNA and miRNA-ncRNA^16^. In this study, we utilized ENCORI to predict the upstream potential miRNAs and lncRNAs of MTHFD2. In addition, we used it to assess the association between MTHFD2, miRNAs, and lncRNAs in EC.

### Gene Set Enrichment Analysis

We utilized the LinkedOmics database (http://www.linkedomics.org/login.php) to obtain the co-expression genes of MTHFD2 in EC. Gene Set Enrichment Analysis (GSEA) software and the ClusterProfiler package were used for signaling pathway enrichment analysis of MTHFD2 in EC^17^-^19^. The R package “ClusterProfiler” was used for performing GO enrichment and KEGG pathway analysis of MTHFD2 co-expression genes, finally, the results were visualized by the “ggplot2” R package.

### Protein-protein interaction analysis

The online tool Search Tool for the Retrieval of Interacting Genes/Proteins (STRING)^20^ (https://string-db.org) is a website that includes many consolidated and integrated data on protein-protein interaction (PPI). Hence, we utilized STRING to explore the PPI of MTHFD2-binding proteins. Finally, 50 MTHFD2-binding proteins were found and the Venn Diagram package was used to screen the MTHFD2 express-related genes interacting with DEGs.

### Cell Culture

The EC cell line (HEC-1A and Ishikawa cells) and HEK293T cells were obtained from Medical Science Research Center, Zhongnan Hospital of Wuhan University, and maintained in DMEM/HIGH GLUCOSE or MEM with NEAA 1% supplemented with 10% fetal bovine serum, and the 1X penicillin-streptomycin solution (Biosharp, China). Cells were cultured in a humidified incubator at 37℃, under 5% CO2.

### Transfection

RNAs (shRNAs) targeting lncRNA SNHG3 and negative control(shNC) were ordered from Tsingke Biotechnology (Wuhan, China). The shSNHG3 sequence was: 5'-GGGCACTTCGTAAGGTTTAAA-3'. MiR-455-5p inhibitor (miR inhibitor) and its negative control (miR-NC) were purchased from RiboBio (Guangzhou, China). Lipofectamine 3000 (Invitrogen, USA) was used according to the manufacturer for cell transfections. The short hairpin RNAs (shRNAs) targeting the mRNA sequence of MTHFD2 (shMTHFD2) were established into a pLKO.1 puromycin vector, the sequence of shMTHFD2 was CCGGCGAATGTGTTTGGATCAGTAT. And the pLKO.1 puromycin vector was used as a negative control shRNA (shNC). The shRNAs were packaged to lentivirus and transfected to HEC-1A and Ishikawa cells, finally, the MTHFD2 knockdown cells were selected by puromycin and verified by western blot analysis.

### Western blot assay

Samples of MTHFD2 knockdown and control EC cells were lysed with RIPA buffer protease inhibitor cocktail (Beyotime, China). After quantified by BCA Protein Assay Kit (Beyotime, China), 30 mg total protein was separated by 10% SDS-PAGE, transferred onto 0.45 µm PVDF membranes, blocked and immunoprobed. Finally, the signals were detected by the chemiluminescence kit (Beyotime, China).

### CCK8 assay

EC shMTHFD2 and shNC cells were seeded at a density of 2000 cells into 96-well plates per well. CCK-8 reagent (Biosharp, China) was added to each well and incubated at 0, 1, 2, 3, 4 and 5 days for 2 hours. The cell viability was assessed by the absorbance (OD) at 450 nm.

### Colony formation assay

For colony formation assay, 500 shMTHFD2 and shNC EC cells were seeded into 6-well plates per well. When the colonies were visible with the naked eye, they were fixed and stained with 0.3% crystal violet in ethanol for 15 minutes.

### Transwell migration assay

For the transwell assay, cells were washed with PBS three times and seeded onto the upper transwell chambers, then cultured in 100 μL medium without FBS. The chambers were placed to 24-well plates with 800 μL medium containing 20% FBS. After 36 hours, the migrated cells were stained with 0.3% crystal violet in ethanol for 15 minutes.

### Co-Immunoprecipitation (co-IP) assay

HEC-1A and Ishikawa cells were collected and lysised by using cell lysis buffer for Western and IP (Beyotime, China) supplemented with a protease inhibitor cocktail ((Beyotime, China). Then, protein G (MCE, China) and normal IgG antibody (Proteintech, China) were used to block the lysate. Next, the lysate was incubated with MTHFD2 antibody (Proteintech, China) or normal IgG antibody (Proteintech, China) overnight at 4°C and then incubated with protein G Sepharose (GE Healthcare, UK) at 4°C for 3 h to conduct the co-IP assay. The immunoprecipitate was denatured at 100°C for 5 min in sample buffer.

### RNA extraction and quantitative real-time PCR

RNA extracting solution (Servicebio, China) was used to extract total RNA from transfected cells. Quantitative real-time PCR (qRT-PCR) was performed according to Taq Pro Universal SYBR qPCR Master Mix kit (Vazyme, China) and miRNA Universal SYBR qPCR Master Mix kit (Vazyme, China). Relative sequences of the primers were listed in Table S1. β-actin or U6 small nuclear RNA was applied as internal control.

### Dual-luciferase reporter assay

All the vectors for dual-luciferase reporter assay were purchased from Genechem. Renilla vector, lncRNA SNHG3 wild-type (WT) or mutant-type (Mut) vectors were framed and co-transfected, respectively, with miR-455 and miR-NC to EC cells. In the same way, Renilla vector and MTHFD2 WT&Mut were respectively co-transfected with miR-455 and miR-NC to EC cells. Dual-Luciferase Reporter Assay System kit (Beyotime) was used to measure the luciferase activity of different groups after 48h.

### Statistical Analyses

All statistical analyses in this study were performed using R, and ROC curves were performed to explore MTHFD2 cutoff values through pROC packages. GraphPad Prism 9.1.2 was applied for the data analyses. Two group comparisons were assessed by Student's t-test, and the significance of the data of multiple experimental groups was assessed by one-way ANOVA. *P < 0.05, **P < 0.01 and ***P < 0.001 were regarded significant, and ns, *P*>0.05 was regarded no significant difference.

## Results

### MTHFD2 was up-regulated in EC

**Figure 2:**
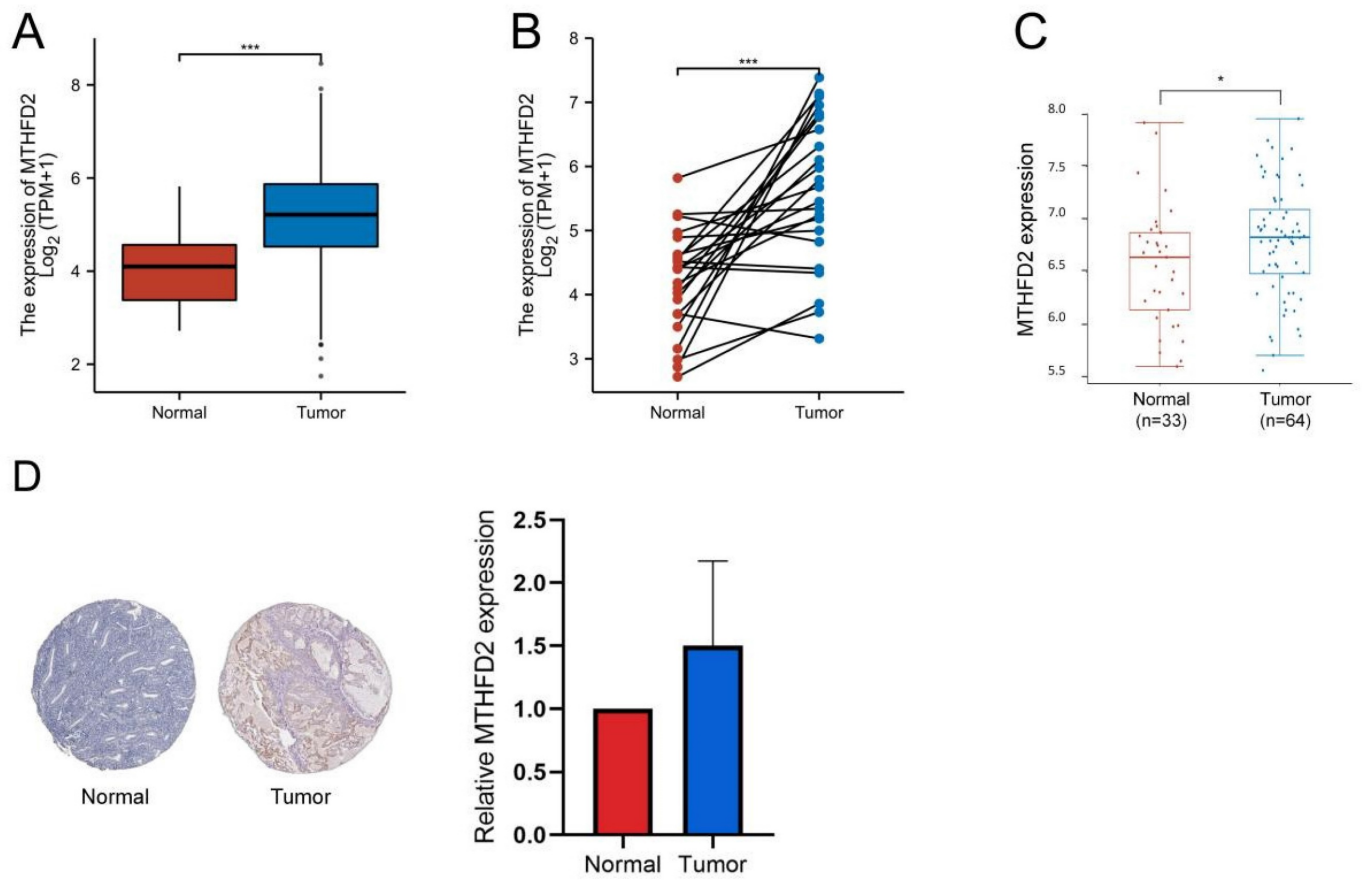
MTHFD2 was up-regulated in EC. (A-C) MTHFD2 mRNA expression levels in endometrial carcinoma samples and adjacent normal samples based on the TCGA and GEO database. (D) MTHFD2 protein level was determined by HPA. The data are presented as mean ± SD.

Firstly, we analyzed the MTHFD2 mRNA expression level in TCGA database. In both the unpaired samples, containing 35 adjacent normal tissues and 552 tumor tissues, and 23 paired samples, the MTHFD2 mRNA level in tumor samples is significantly increased than that in adjacent normal samples (Fig. 2A, B). The results based on GEO datasets have also confirmed that MTHFD2 mRNA was significantly up-regulated in EC samples (Fig. 2C). To further evaluate the MTHFD2 protein level, we applied HPA, and results also indicated that MTHFD2 was up-regulated in endometrial carcinoma (Fig. 2D).

### Up-regulation of MTHFD2 was correlated with adverse clinical parameters in EC

**Figure 3:**
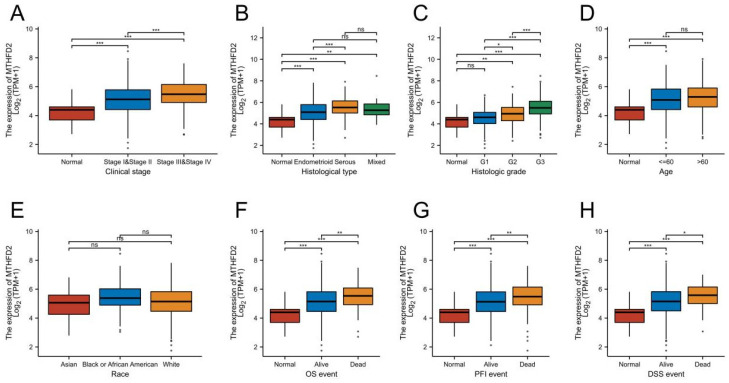
Up-regulation of MTHFD2 was correlated with adverse clinical parameters in EC. The correlation analysis of MTHFD2 expression and clinical parameters, including (A) clinic stage, (B) histological type, (C) histologic grade, (D) age, (E) Race, (E-H) OS, PFI, and DSS event. The data are presented as mean ± SD.

To make clear the clinic significance of MTHFD2 expression level, we conducted the relationship of MTHFD2 level with clinic stage, histological type, histologic grade, age, OS event, progression-free interval (PFI), and disease-specific survival (DSS) event. These results showed that the up-regulation of MTHFD2 in EC was significantly associated with adverse clinic stage, histological type, histologic grade, and poor OS, PFI, and DSS (Fig. 3A-C, F-H). In addition, its expression level was unrelated to age and race (Fig. 3D, E).

### High MTHFD2 expression was correlated with a poor prognosis

**Figure 4:**
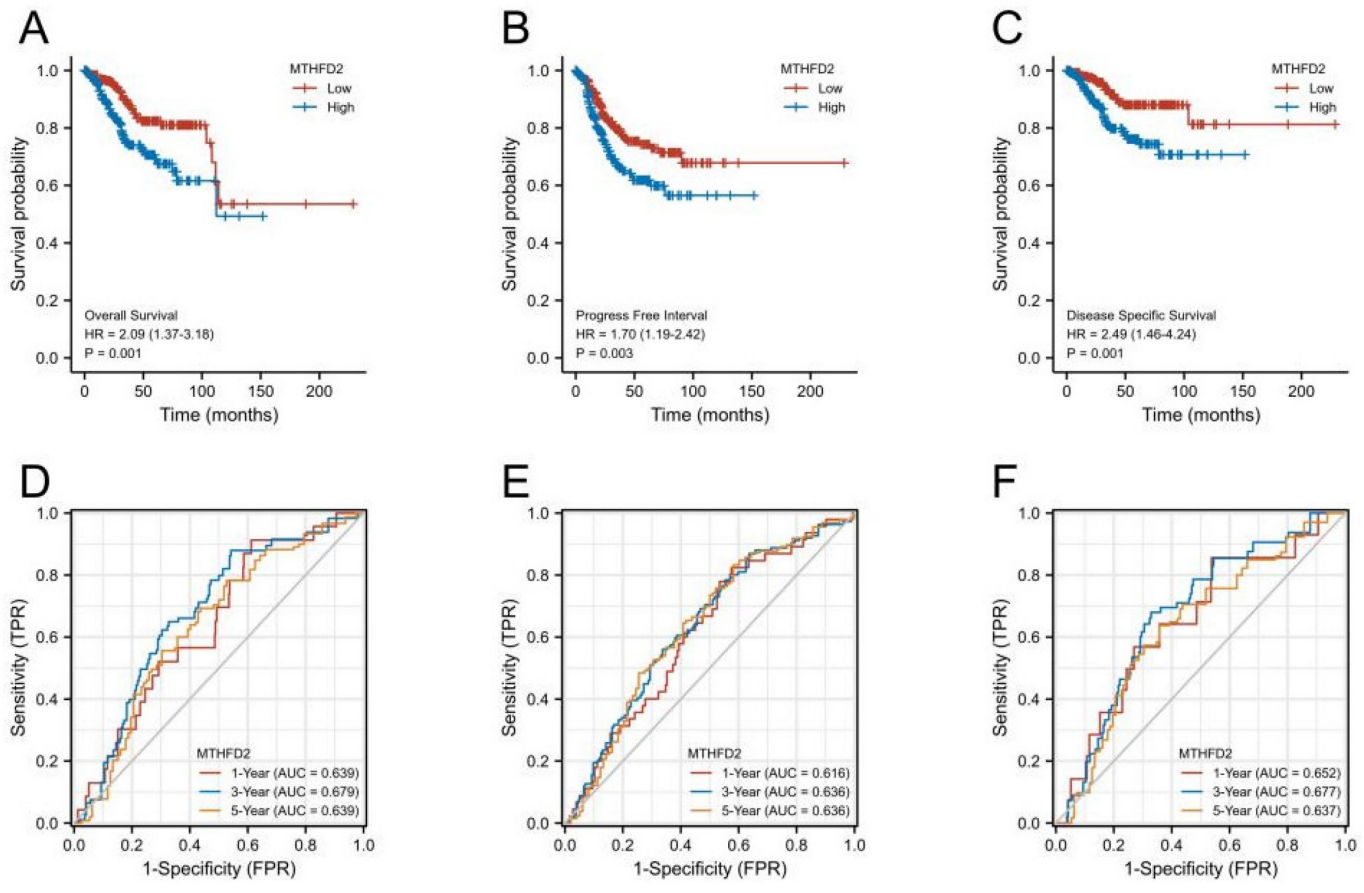
High MTHFD2 expression was correlated with poor prognosis. (A-C) Kaplan-Meier survival curves analyze the association between MTHFD2 level and endometrial carcinoma patients' OS, PFI, DSS and based on the TCGA-UCEC database. (D-F) The diagnostic value of MTHFD2 in endometrial carcinoma was determined by time-dependent ROC curves.

Next, we performed KM survival and log-rank test to analyze the association between MTHFD2 mRNA level with patients' prognosis (OS, PFI, and DSS) of EC in the TCGA. Figures 4A-C showed that EC patients with higher MTHFD2 expression had a relatively poor prognosis compared to those with a lower MTHFD2 level (OS, PFI, and DSS). The time-dependent ROC prognosis analysis showed that the MTHFD2 mRNA level was good at predicting 1-year (AUC = 0.639), 3-year (AUC = 0.679), and 5-year (AUC = 0.639) OS of EC patients (Fig. 4D). Its expression level also showed a comparatively good performance in the prediction of 1-year (AUC = 0.616), 3-year (AUC = 0.636), and 5-year (AUC = 0.636) PFI and 1-year (AUC = 0.652), 3-year (AUC = 0.677), and 5-year (AUC = 0.637) DSS of EC patients (Fig. 4E, F).

### Prognostic potential of the MTHFD2 level based on clinical subgroups

**Figure 5:**
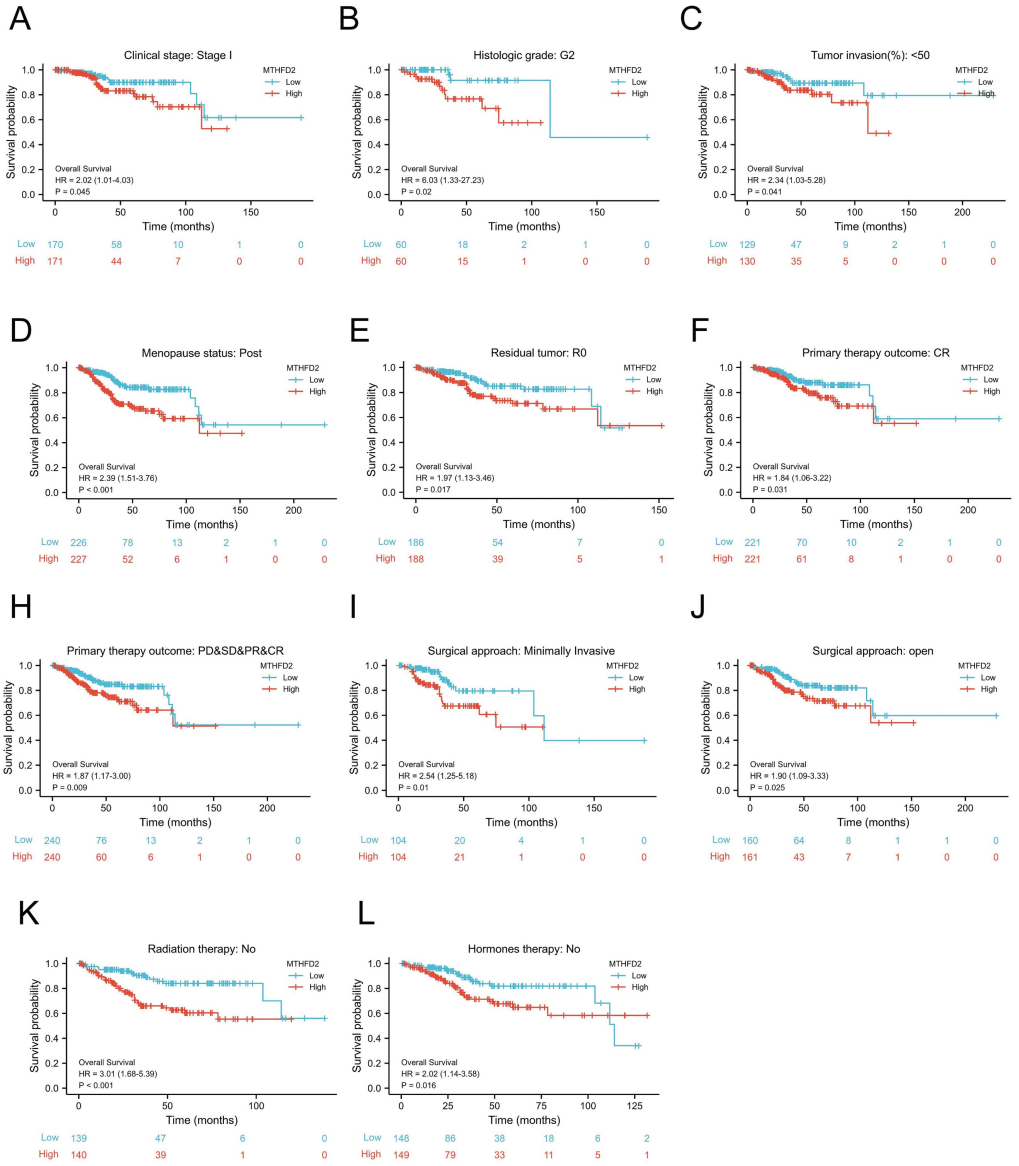
Prognostic potential of the MTHFD2 level based on clinical subgroups. (A) Clinical stage: stage I, (B) histologic grade: G2, (C) tumor invasion(%)˂50, (D) menopause status: post, (E) residual tumor: R0, (F) primary therapy outcome: CR, (G) primary therapy outcome: PD&SD&PR&CR, (I) surgical approach: minimally invasive, (J) surgical approach: open, (K) radiation therapy: no, (L) hormones therapy: no.

To further evaluate the prognostic role of MTHFD2 in EC, we next performed a correlation analysis of MTHFD2 expression and OS across several subgroups by different clinical features. Consistent with the overall prognosis analysis above, the high MTHFD2 expression group was linked to a poor prognosis compared to the low MTHFD2 expression group, including the subgroup of Clinical stage: stage I, histologic grade: G2, tumor invasion(%)˂50, menopause status: post, residual tumor: R0, primary therapy outcome: CR, PD&SD&PR&CR, surgical approach: minimally invasive, surgical approach: open, radiation therapy: no, hormones therapy: no (Fig. 5A-L).

### Gene function and related pathway enrichment analysis

**Figure 6:**
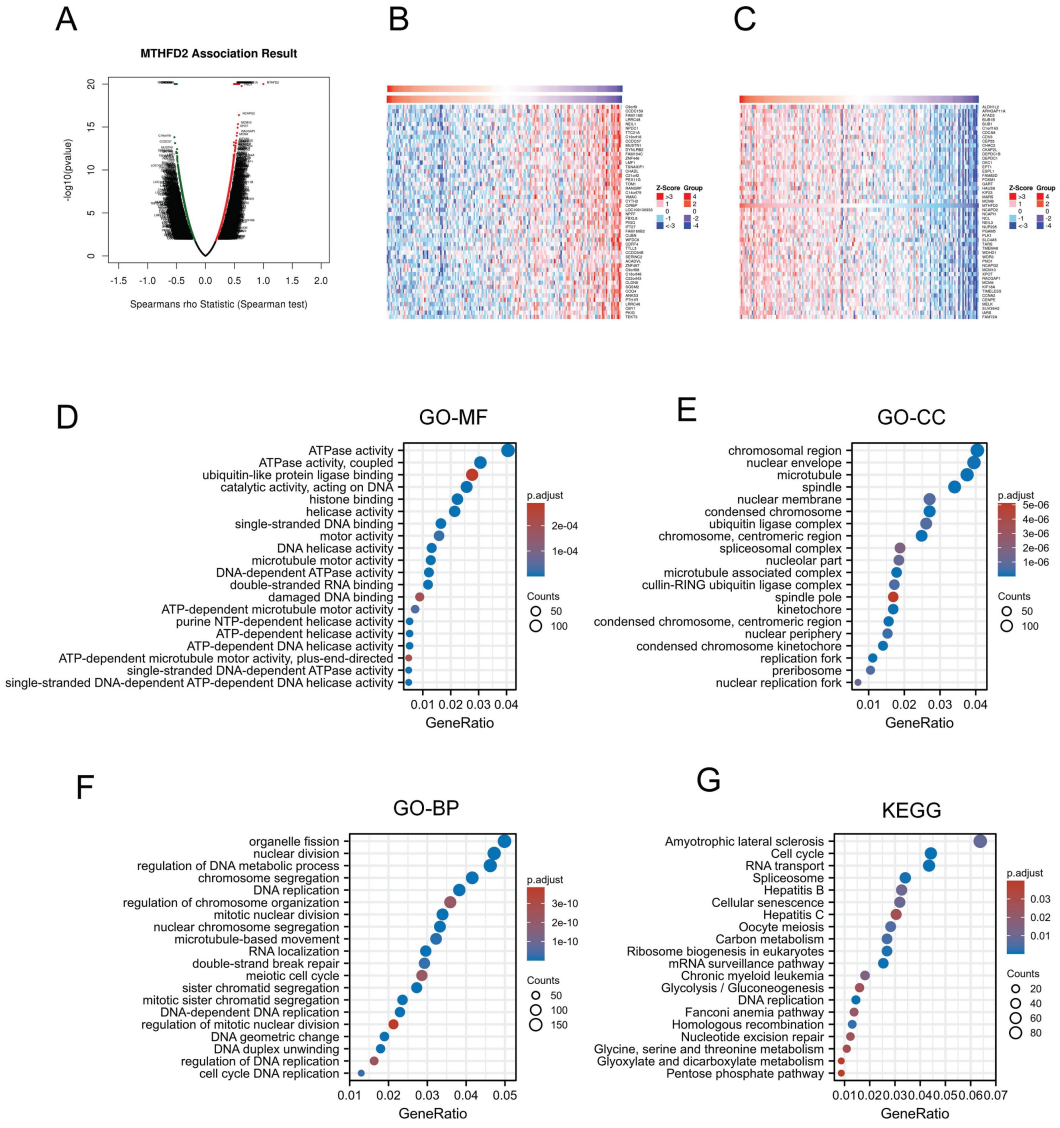
Gene function and related pathway enrichment analysis. (A-C) The association between MTHFD2 expression and its top 100 co-expressed gene network. (D-G) GO and KEGG analysis based on these co-expressed genes.

Previous analyzes have shown that MTHFD2 played a vital role in the prognosis of EC patients. Thus, we applied LinkedOmics to screen the significant positive association with the MTHFD2 genes (Fig. 6A). 50 positively correlated genes and 50 negatively correlated genes were shown below (Fig. 6B, C). Then the GO analysis and KEGG analysis were used to study the functions of MTHFD2. Molecular function (MF) of MTHFD2 showed that relative genes were correlated with helicase activity, ATPase activity, and DNA-dependent ATPase activity (Fig. 6D) (Table 1). GO analysis of cellular component (GO-CC) and the biological process (GO-BP) demonstrated that MTHFD2 was involved in the chromosomal region, nuclear envelope, DNA replication, organelle fission, nuclear division, regulation of DNA metabolic process (Fig. 6E, F) (Table 2, 3). KEGG molecular pathways were enriched in amyotrophic lateral sclerosis, cell cycle, RNA transport, and spliceosome (Fig. 6G) (Table 4).

### MTHFD2-related signaling pathways based on GSEA

**Figure 7:**
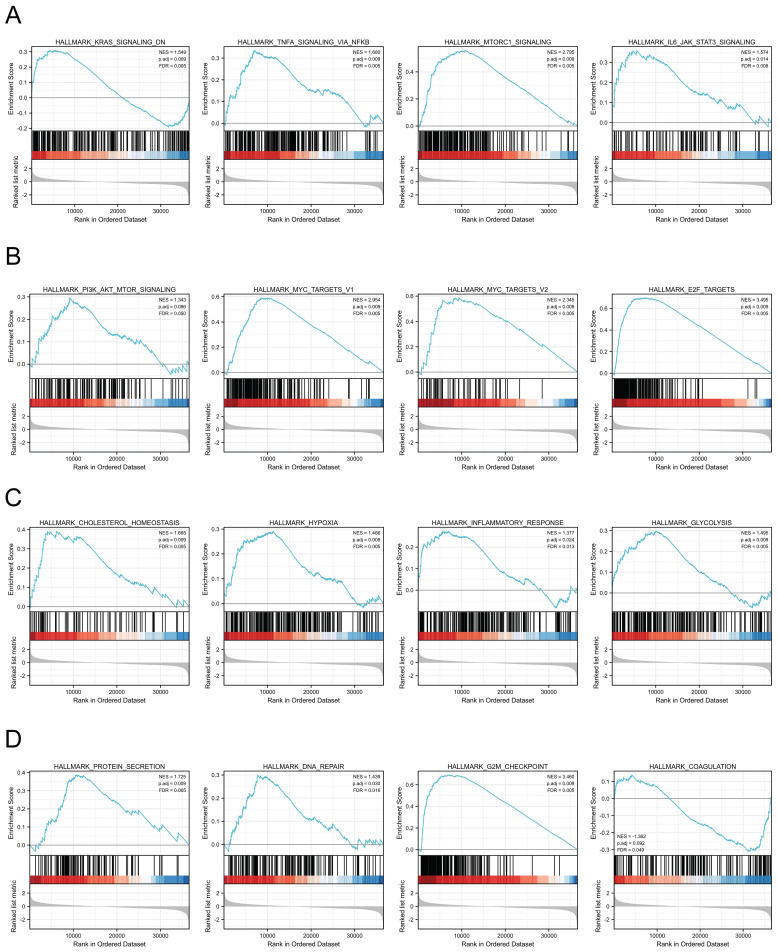
MTHFD2-related signaling pathways based on GSEA. (A-D) Analysis of MTHFD2-related signaling pathways by GSEA software.

We next assessed the functions of MTHFD2 by analyzing the differentially expressed genes between the low and high MTHFD2 expression level groups based on the MTHFD2 median expression value. GSEA demonstrated that relative DEGs were enriched in the KRAS signaling pathway, TNFα signaling via NF-kB, MTORC1 signaling pathway, IL6-JAK-STAT3 signaling pathway, PI3K-AKT-MTOR signaling, MYC targets v1, MYC targets v2, E2F targets, cholesterol homeostasis, hypoxia, inflammatory response, glycolysis, protein secretion, DNA repair, G2M checkpoint, coagulation (Fig. 7).

### The relationship between MTHFD2 and immune infiltration and immune checkpoints in EC

**Figure 8:**
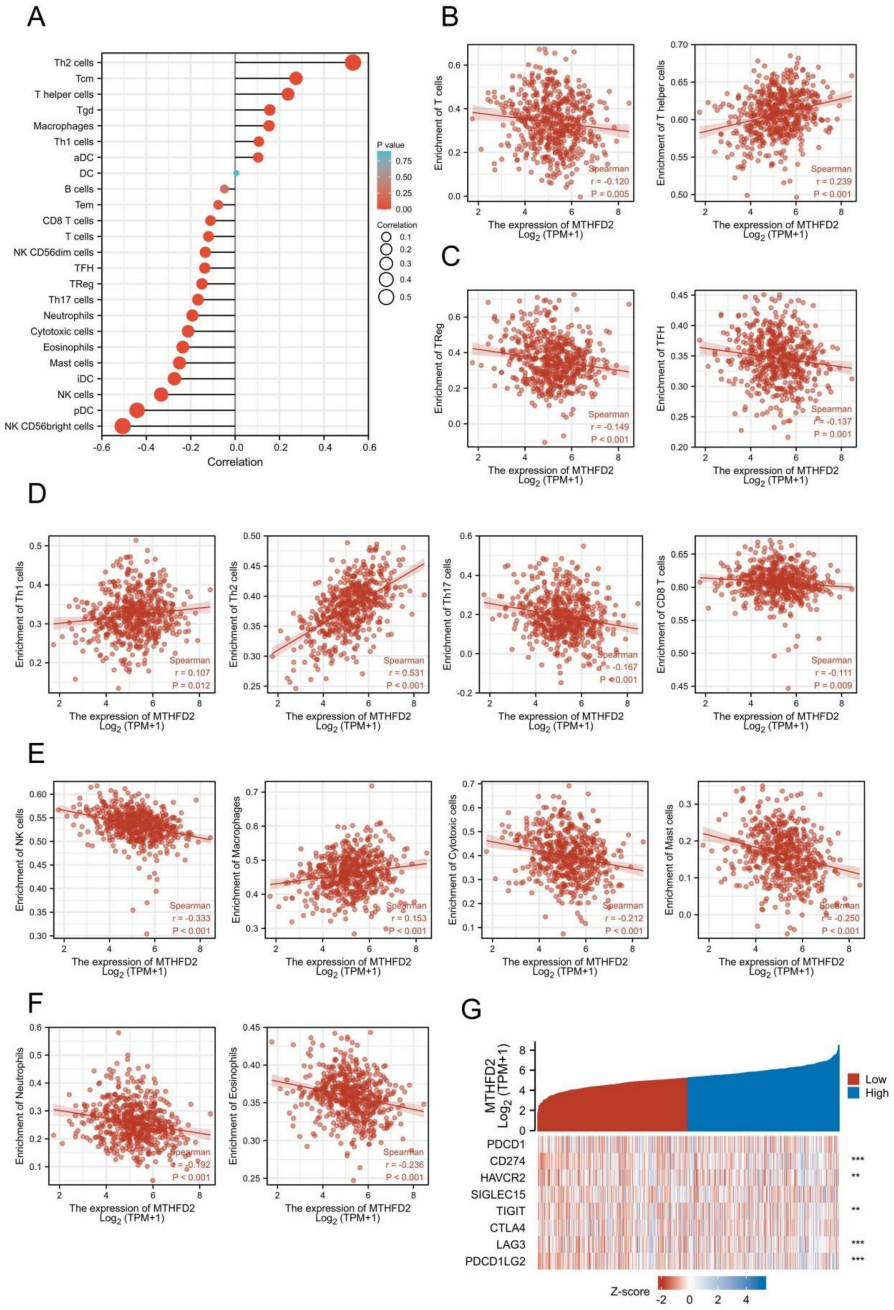
The relationship between MTHFD2 and immune infiltration and immune checkpoints in EC. (A-F) The association between MTHFD2 expression level and immune cell infiltration level. (G) Correlation analysis of MTHFD2 level and immune checkpoint-related genes in EC.

Increasing evidence has demonstrated the important role of immune infiltration in cancer progression and patients' prognosis. Hence, we evaluated the association between the MTHFD2 expression and the immune infiltration level in EC. The results showed that MTHFD2 was positively related to Th2 cells, Tcm, T helper cells, Tgd and Macrophages. While it had a negative association with NK CD56bright cells, pDC, NK cells, iDC and mast cells (Fig. 8A-F). Considering that MTHFD2 might be a potential oncogene in EC, the association of MTHFD2 with PDCD1, CD274 (PD-L1), HAVCR2, SIGLEC15, TIGIT, CTLA4, LAG3, and PDCD1LG2 (PD-L2) in EC was explored. As a consequence, MTHFD2 expression was significantly related to CD274, HAVCR2, TIGIT, LAG3, and PDCD1LG2 in EC (Fig. 8G).

### Correlation between MTHFD2 expression and OS in different immune infiltration subgroups of EC

**Figure 9:**
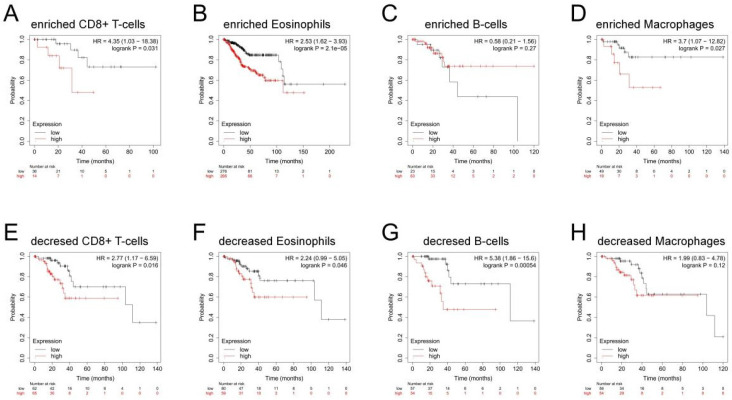
Correlation between MTHFD2 expression and OS in different immune infiltration subgroups of EC. (A-H) Kaplan-Meier plotter analyzed the correlations between MTHFD2 expression and OS based on different immune cell subgroups in EC patients.

In the previous analysis, MTHFD2 showed an important role in EC patients' prognosis and a close association with the immune infiltration of EC. Therefore, there may be a hypothesis that MTHFD2 may affect the prognosis of EC patients partly through immune infiltration. KM plotter analysis of MTHFD2 expression in EC following CD8+ T cells, eosinophilic cells, B cells, and macrophages was conducted. We found that higher MTHFD2 levels associated with enriched CD8+ T cells, eosinophilic, and macrophage cells had a worse prognosis (Fig. 9). These results indicated the correction between immune infiltration and high MTHFD2 expression and the potential mechanism that MTHFD2 affected the prognosis of EC patients via immune infiltration.

### Knockdown of MTHFD2 inhibited malignant phenotype of EC cells

**Figure 10:**
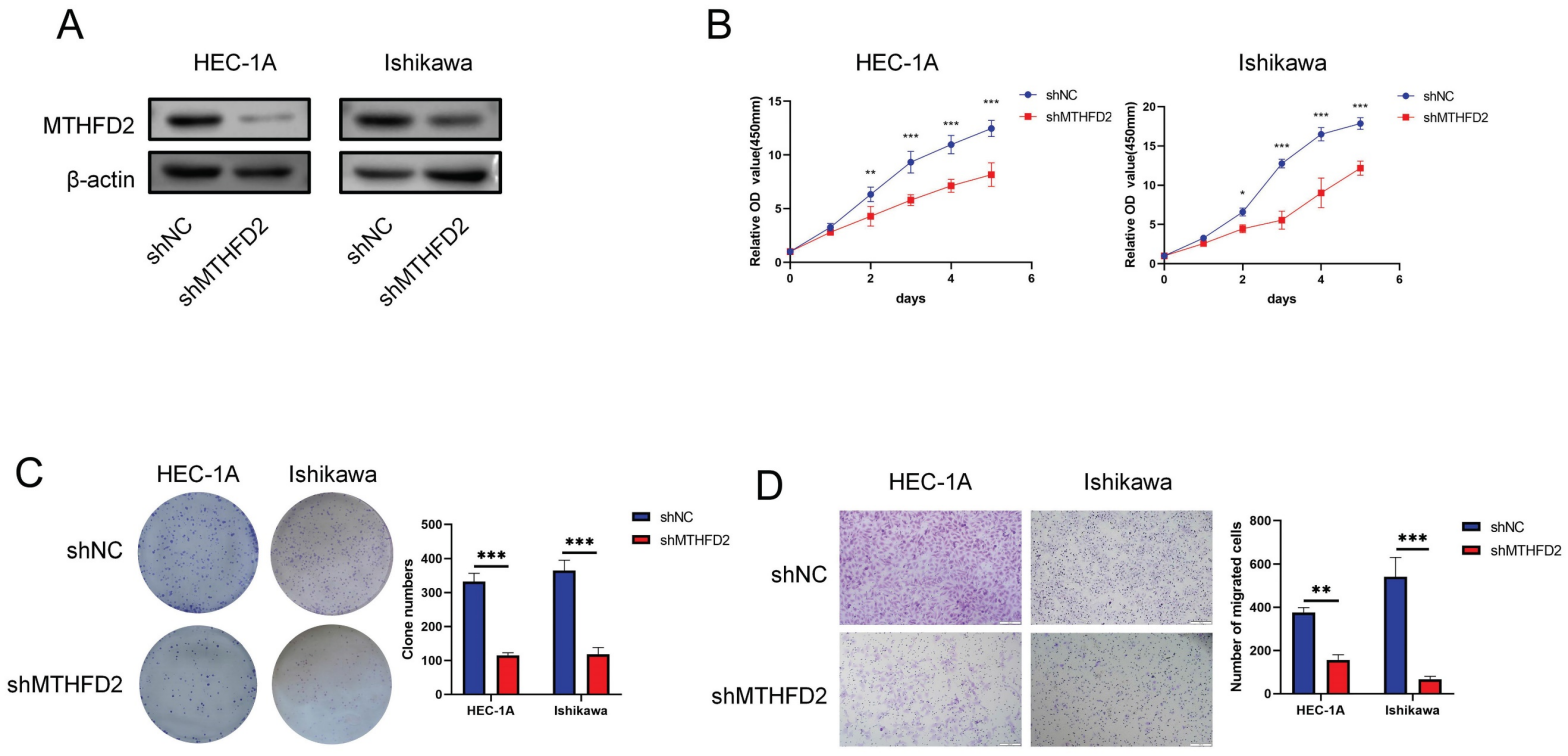
Knockdown of MTHFD2 inhibited malignant phenotype of EC cells (A) Western blotting was conducted to verify the knockdown efficacy of MTHFD2 in HEC-1A and Ishikawa cells. (B) CCK8 assay assessed the proliferation of HEC-1A and Ishikawa cells transfected with shMTHFD2 and shNC. (C) The representative images of colony formation assay, and statistical analysis was shown behind. (D) The representative images of transwell migration assays of HEC-1A and Ishikawa cells transfected with shMTHFD2 and shNC(scale bar 200㎛). The data are presented as mean ± SD.

The analysis above indicated the important role of MTHFD2. Hence, we conducted relative experiments to validate it. First, we established the MTHFD2 knockdown HEC-1A and Ishikawa cells and verified by Western blot analysis (Fig. 10A). The CCK8 assay showed that knockdown of MTHFD2 significantly inhibited the proliferation, colony formation and migration of HEC-1A and Ishikawa cells (Fig. 10B-D).

### Creating protein interaction networks

**Figure 11:**
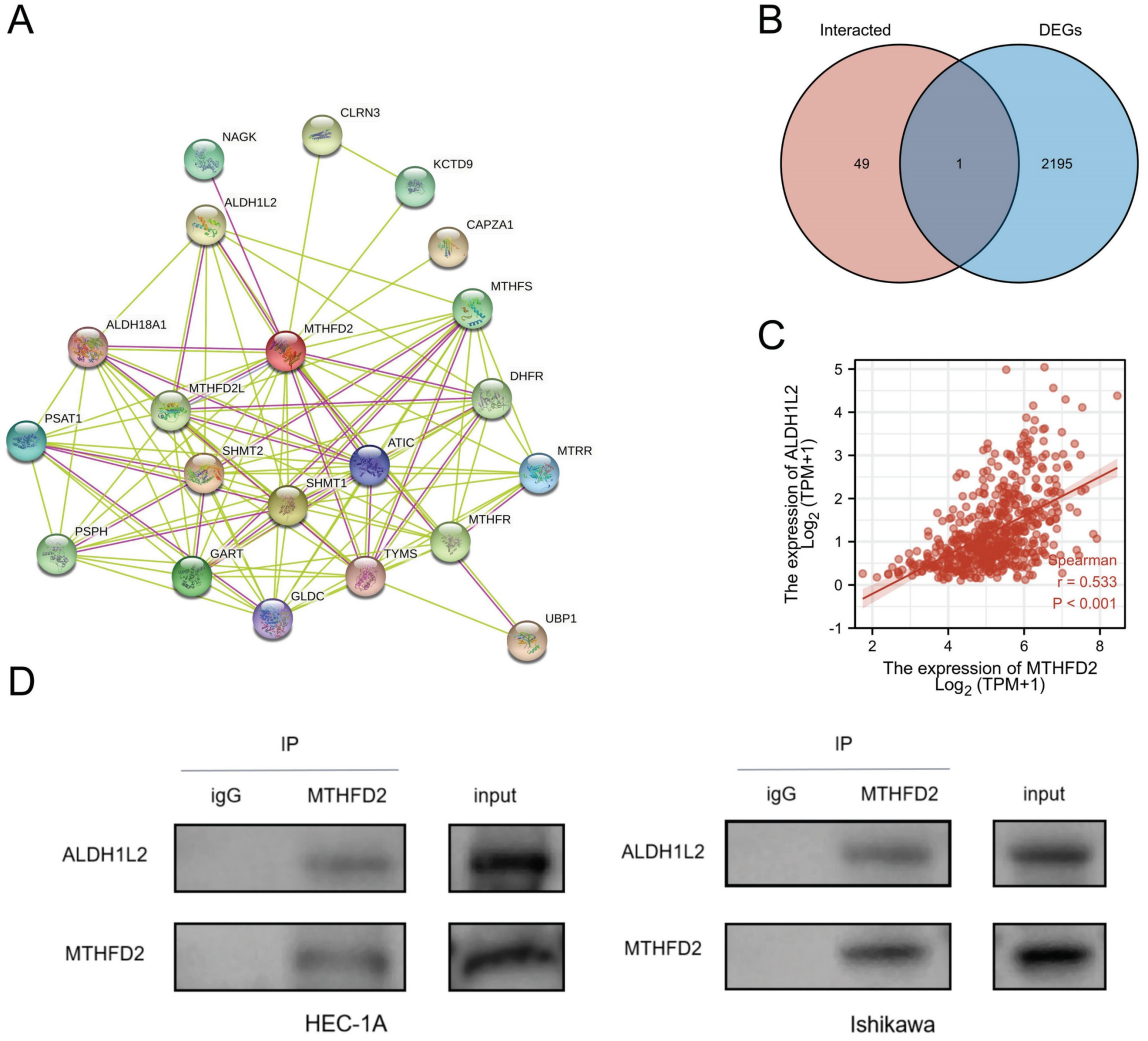
Creating protein interaction networks. (A) Protein interaction of MTHFD2, (B) Venn diagram showed the MTHFD2-interacted proteins with MTHFD2 expression-correlated DEGs, (C) Correction expression between MTHFD2 and ALDH1L2. (D) MTHFD2 antibody or normal rabbit IgG antibody were subjected to CO-IP, then the indicated antibodies were used for immunoblotting analysis in HEC-1A and Ishikawa cells transfected with shMTHFD2 and shNC.

Protein-protein interaction (PPI) has also been stressed in many studies due to its important effect on tumor progression as PPI regulates protein's activity. Hence, we analyze the MTHFD2 protein PPI network by the STRING. Fig. 11A showed the 50 proteins which had interaction with MTHFD2 protein. To find out the key protein that interacted with MTHFD2 protein in EC, the Venn diagram was used to compare MTHFD2-interacted proteins with MTHFD2 expression-correlated DEGs. As a result, ALDH1L2 was screened out (Fig. 11B). In addition, ALDH1L2 had a remarkable positive association with MTHFD2 (r = 0.533, P < 0.001) (Fig. 11C). And Co-IP confirmed the interaction between MTHFD2 and ALDH1L2 in HEC-1A and Ishikawa cells (Fig. 11D). These results indicated that the interaction of MTHFD2 and ALDH1L2 might affect the progression of EC.

### Exploration of the upstream miRNAs of MTHFD2

**Figure 12:**
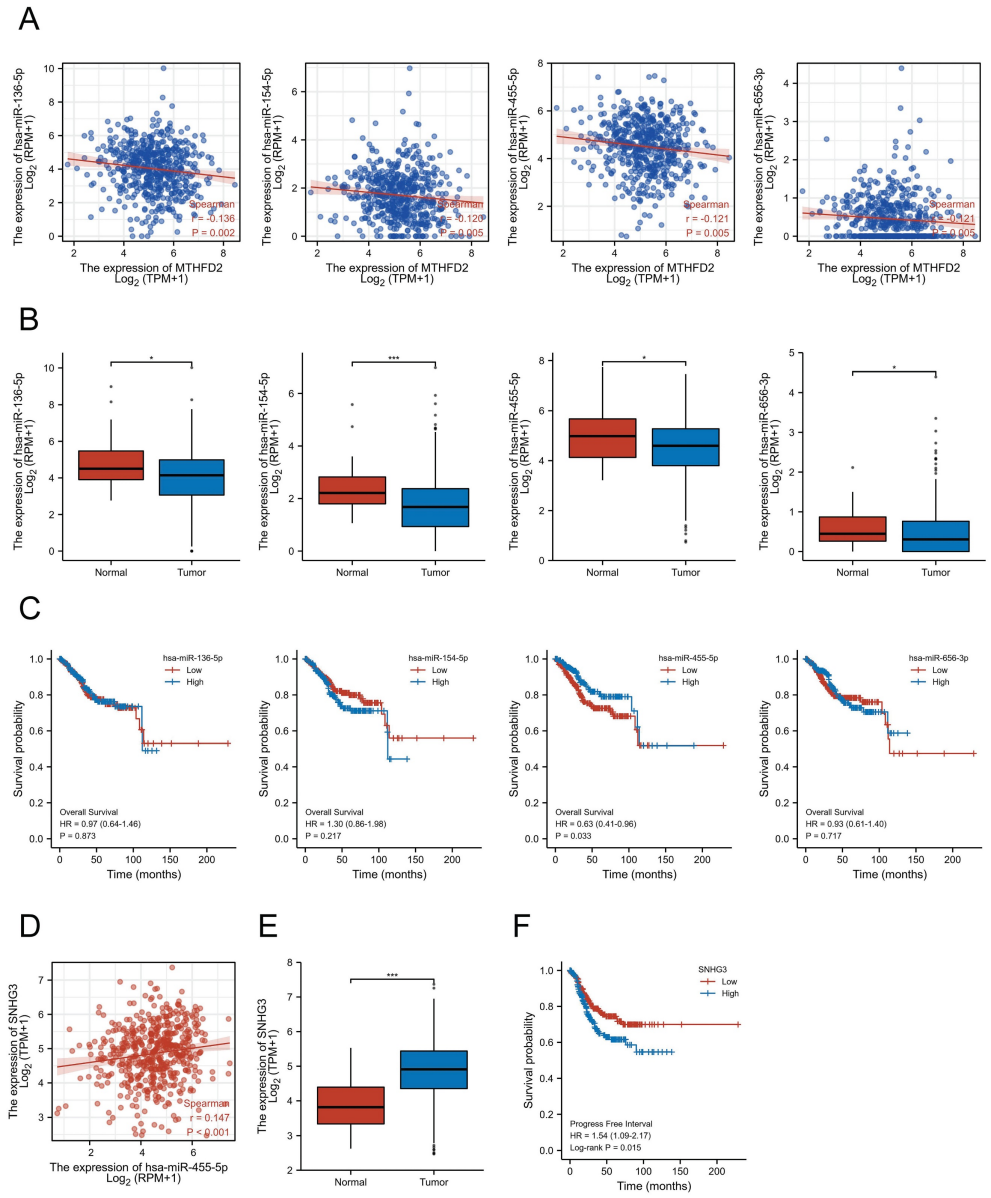
SNHG3/hsa-miR-455-5p/MTHFD2 regulatory network. (A) Analysis of the association between MTHFD2 expression and hsa-miR-136-5p, hsa-miR-154-5p, hsa-miR-455-5p, and hsa-miR-656-3p in TCGA-UCEC, (B) Analysis of hsa-miR-136-5p, hsa-miR-154-5p, hsa-miR-455-5p, and hsa-miR-656-3p expression level based on TCGA database, (C) Correction between hsa-miR-136-5p, hsa-miR-154-5p, hsa-miR-455-5p, and hsa-miR-656-3p expression level and the prognosis of EC patients, (D) Correction expression between SNHG3 and hsa-miR-455-5p, (E) SNHG3 expression level in EC, (F) Prognosis analysis of SNHG3 expression in EC based on TCGA database.

Accumulating studies have revealed the important role of ncRNAs in regulating gene expression in various cancers^21^. To explore whether MTHFD2 was modulated by some ncRNAs, we first screened the possible upstream miRNAs that might regulate MTHFD2, and finally, 139 miRNAs were found (Supplementary Table 1). As upstream miRNAs negatively regulated the gene post-transcriptional level, 4 miRNAs, including hsa-miR-136-5p, hsa-miR-154-5p, hsa-miR-455-5p, and hsa-miR-656-3p, were found to be negatively related to MTHFD2 expression level and differently expressed in EC based on TCGALUAD database (Fig. 12A, B). Further prognosis analysis showed only hsa-miR-455-5p level was related to patients' prognosis (Fig. 12C). Taken together with these analyses in this part, hsa-miR-455-5p was screened as the most likely regulatory upstream miRNA of MTHFD2 in EC.

### Exploration of potential upstream lncRNAs of hsa-miR-455-5p

To explore upstream lncRNAs regulating hsa-miR-455-5p, ENCORI database was applied and 16 candidate lncRNAs that interacted with hsa-miR-455-5p were founded (Supplementary Table 2). Through correction analysis, expression analysis, and survival analysis, SNHG3 was found to be the only lncRNA that correlated to hsa-miR-455-5p expression level and was highly expressed in EC (Fig. 12D, E). Besides, patients with higher SNHG3 expression level was associated with a worse prognosis (Fig. 12F).

### Validation of SNHG3/hsa-miR-455-5p/MTHFD2 in EC cells

**Figure 13:**
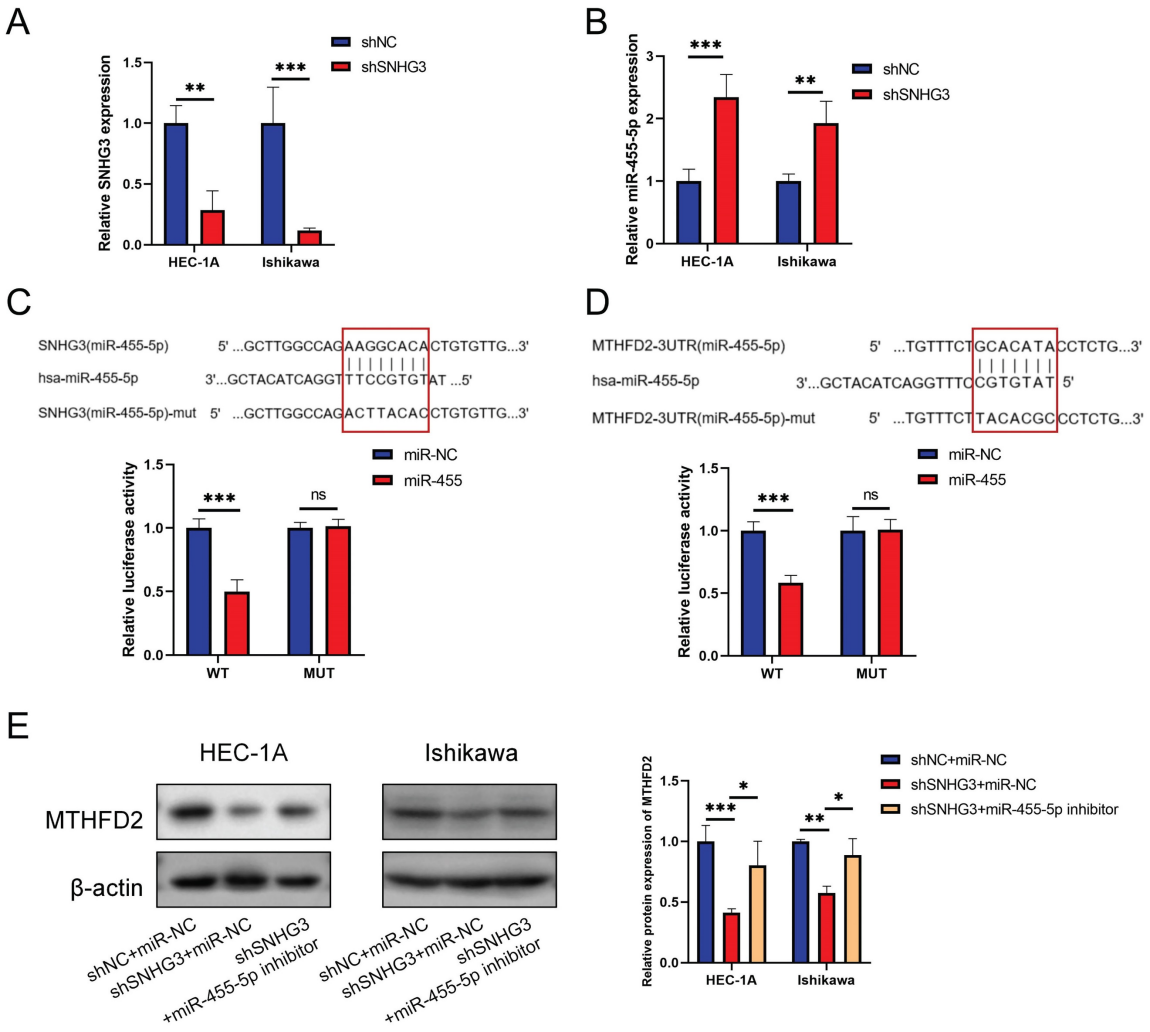
Validation of SNHG3/hsa-miR-455-5p/MTHFD2 regulation axis in EC cells. (A-B) Relative SNHG3 (A) and miR-455-5p (B) expression in HEC-1A and Ishikawa cells transfected with shSNHG3 and control by qRT-PCR. (C-D) Potential binding sites of SNHG3/hsa-miR-455-5p (C) and hsa-miR-455-5p/MTHFD2 (D), and dual-luciferase assay validated their binds in HEC-1A cells. (E) Western blotting tested the MTHFD2 expression in HEC-1A and Ishikawa cells transfected with shSNHG3 or shSNHG3+ miR-455-5p inhibitor, and statistical analysis was shown behind.

To make the SNHG3/hsa-miR-455-5p/MTHFD2 axis more credible, we performed relative experimental verification in *vitro*. Fig. 13A validated the effcience of shSNHG3 in EC cells, and Fig. 13B demonstrated that knockdown of SNHG3 enhanced expression of miR-455-5p. We conducted the luciferase activity was also decreased when co-transfection of lncRNA SNHG3-WT and miR-455 (Fig. 13C), then the dual-luciferase reporter assay results showed that co-transfection of MTHFD2-WT and miR-455 significantly inhibited luciferase activity than control group (Fig. 13D). Finally, we evaluated the protein level of MTHFD2 by western blot assay. The result demonstrated that hsa-miR-455-5p inhibitor rescued the decrease of MTHFD2 caused by knockdown of SNHG3 (Fig. 13E).

## Discussion

Myometrial invasion, histotype and lymph vascular space invasion have been stressed in the management and prognosis in EC^21^. Recent years, molecular subtypes were also found to play an important role in EC, for example, Tumor Protein 53 (TP53) mutations, accompanied by abnormal p53 expression (p53-abn) indicate the worst prognosis^22^. In the present study, we analyzed the MTHFD2 expression level in EC and found that both MTHFD2 mRNA and protein expression levels were up-regulated in tumor tissues compared with normal adjacent tissues. In addition, the high MTHFD2 level was associated with adverse clinical features, including clinic stage, histological type, histologic grade, OS event, PFI, and DSS event. KM curves indicated the potential diagnostic biomarker of MTHFD2 as its expression level was linked to patients' OS, PFI, and DSS based on the TCGA database. Further correction analysis demonstrated that MTHFD2 level was associated with tumor immune infiltration. Through predictive analysis, SNHG3/hsa-miR-455-5p axis might be the potential upstream regulation of MTHFD2.

Metabolomics molecular factors show a good prospect in the diagnosis and management of EC. Jacopo et al. found that serum metabolomes, including serine, glutamic acid, phenylalanine, and glyceraldehyde 3-phosphate were lower in EC patients^23^. A systmetic review relaved that metabolomics was suitable for a non-invasive diagnosis and predict tumor behavior of EC^24^, MTHFD2 is a key enzyme in mitochondria one-carbon metabolism and takes part in several important substance catalyzes, including serine and glycin converting to TMP and 10-formyltetrahydrofolic acid. Recently, accumulating studies have indicated the important role of MTHFD2 in tumor progression. MTHFD2 was found up-regulated in many cancers and the high MTHFD2 expression level was related to a poor prognosis^8^-^11^. Consistent with the previous studies, the MTHFD2 mRNA expression level was significantly upregulated in EC and the high MTHFD2 level was linked to adverse clinical features and poor prognosis in the present study. Based on the HPA database, the MTHFD2 was also higher in tumor tissues at the protein level. Unsatisfactorily, the difference was not significant even though the trend was consistent, this was most likely because the sample size was too small. To reach a more reliable conclusion, an analysis containing more samples is necessary.

Previous studies revealed the role of MTHFD2 in tumor progress and the mechanism that MTHFD2 affected cancers^8^, ^12^. Therefore, we investigated the biological function of MTHFD2 and found that it was mainly associated with the cell cycle, DNA replication, nuclear division and regulation of DNA metabolic process. Moreover, GSEA enrichment confirmed that MTHFD2 was significantly related to the KRAS signaling pathway, TNFα signaling via NF-kB, MTORC1 signaling pathway, IL6-JAK-STAT3 signaling pathway, MYC targets v1, MYC targets v2, E2F targets, cholesterol homeostasis, hypoxia, inflammatory response, glycolysis, protein secretion, DNA repair, G2M checkpoint, coagulation. These results demonstrated that MTHFD2 might affect EC progress in various parts and be involved in many important pathways.

Protein-protein interaction (PPI) has also been stressed in many studies due to its important effect on tumor progression as PPI regulates protein's activity^25^. In our study, we screened ALDH1L2 as the most potential interaction protein of MTHFD2. ALDH1L2 and MTHFD2 are both glycine and serine fuel one carbon metabolism enzymes. ALDH1L2 encodes Mitochondrial 10-fTHF dehydrogenase and plays an important role in providing reduced NADPH in mitochondria^26^. This NADPH production way is necessary for the reduction of oxidized glutathione, hence, ALDH1L2 is important in shifting the ratio of GSH/GSSG. The loss of ALDH1L2 decreases the cellular capacity to maintain redox homeostasis resulting in oxidative stress^27^.

Immune infiltration can affect the tumor immune escape and tumor progress. A previous study has indicated that MTHFD2 induced cancer immune evasion^28^. In the present study, the MTHFD2 level showed a significant correction with the immune infiltration level in EC. More specifically, MTHFD2 was positively related to Th2 cells, Tcm, T helper cells, Tgd and Macrophages. While it had a negative association with NK CD56bright cells, pDC, NK cells, iDC and mast cells. In addition, MTHFD2 expression was confirmed to be significantly related to CD274, HAVCR2, TIGIT, LAG3, and PDCD1LG2 in EC, and the increased MTHFD2 levels associated with enriched CD8+ T cells, eosinophilic, and macrophages cells had a worse prognosis. These results indicated that MTHFD2 affected the prognosis of EC patients partly via immune infiltration, and targeting MTHFD2 might play an important role in tumor immune escape and the processes of EC.

Previous analyses have shown the effect and the possible regulation mechanism of MTHFD2. However, the upstream regulation of MTHFD2 was unclear. Considering the important role of miRNAs in regulating gene expression in various cancers, we finally found hsa-miR-455-5p as the potential upregulation miRNA targeting MTHFD2 in EC via correlation analysis, expression analysis, and survival analysis together. And we also screened SNHG3 as the most potential upstream lncRNA of hsa-miR-455-5p. Taking these two predictable results together, the SNHG3/hsa-miR-455-5p pathway may mediate the high expression of MTHFD2.

## Conclusion

This study demonstrated that the increased MTHFD2 was associated with adverse clinical features, poor prognosis of EC and immune infiltration. Knockdown of MTHFD2 inhibited malignant phenotype of EC cells. SNHG3/hsa-miR-455-5p axis might mediate the increased expression of MTHFD2. This study elucidated the role of MTHFD2 and provide new insights into the diagnosis and treatment of EC.

## Supplementary Material

Supplementary table.

## Figures and Tables

**Table 1:** Molecular function of MTHFD2

Ontology	ID	Description	GeneRatio	p.adjust
MF	GO:0016887	ATPase activity	123/3035	5.24165E-10
MF	GO:0042623	ATPase activity, coupled	93/3035	1.40717E-08
MF	GO:0031625	ubiquitin protein ligase binding	76/3035	0.002775209
MF	GO:0140097	catalytic activity, acting on DNA	78/3035	1.77982E-09
MF	GO:0042393	histone binding	68/3035	2.87899E-07

MF: Molecular Function

**Table 2:** Cellular component of MTHFD2

Ontology	ID	Description	GeneRatio	p.adjust
CC	GO:0098687	chromosomal region	127/3139	3.70922E-18
CC	GO:0005635	nuclear envelope	124/3139	9.26713E-08
CC	GO:0005874	microtubule	118/3139	6.19715E-09
CC	GO:0005819	spindle	107/3139	3.84043E-10
CC	GO:0031965	nuclear membrane	85/3139	7.62029E-07

CC: Cellular Component

**Table 3:** Biological process of MTHFD2

Ontology	ID	Description	GeneRatio	p.adjust
BP	GO:0048285	organelle fission	150/3007	8.44745E-17
BP	GO:0000280	nuclear division	142/3007	1.28303E-17
BP	GO:0051052	regulation of DNA metabolic process	139/3007	1.57216E-14
BP	GO:0006260	DNA replication	115/3007	6.20643E-21
BP	GO:0033044	regulation of chromosome organization	108/3007	2.18929E-10

BP: Biological Process

**Table 4:** MTHFD2 related molecular pathways

Ontology	ID	Description	GeneRatio	p.adjust
KEGG	hsa05014	Amyotrophic lateral sclerosis	88/1379	0.008467296
KEGG	hsa04110	Cell cycle	61/1379	3.42337E-14
KEGG	hsa03013	RNA transport	60/1379	2.50848E-05
KEGG	hsa03040	Spliceosome	47/1379	0.000888248
KEGG	hsa05161	Hepatitis B	45/1379	0.010896886

KEGG: Kyoto Encyclopedia of Genes and Genomes

## Abbreviations

MTHFD2: methylenetetrahydrofolate dehydrogenase 2
KM: kaplan-Meier
ncRNAs: noncoding RNAs
GEO: Gene Expression Omnibus
HPA: human protein atlas
ENCORI: encyclopedia of RNA interactomes
GSEA: Gene Set Enrichment Analysis
STRING: search tool for the retrieval of interacting genes/proteins
PPI: protein-protein interaction
OS: overall survival
PFI: progression-free interval
DSS: disease-specific survival
MF: molecular function
CC: cellular component
BP: biological process
