# Supplementary material for: SNHG3/hsa-miR-455-5p Axis-mediated High Expression of MTHFD2 Correlates with Tumor Immune Infiltration and Endometrial Carcinoma Progression
Source: Int J Med Sci. 2023 Jul 3;20(8):1097–113. doi: 10.7150/ijms.81962 (PMC10357439; doi:10.7150/ijms.81962)
Supplement: Supplementary file 1 — Supplementary table. [file ijmsv20p1097s1.pdf]

Supplementary Table S1. Primers used for Real time PCR

**Target gene**	**Primer (5'-3')**
U6	RT: GTCGTATCGACTGCAGGGTCCGAGGTATTCGCAGTCGATACGACAAAATATG
	F: AGCACATATACTAAAATTGGAACGAT
	Common R: ACTGCAGGGTCCGAGGTATT
miR-455-5p	RT: GTCGTATCGACTGCAGGGTCCGAGGTATTCGCAGTCGATACGACCGATGT
	F: CGGCTATGTGCCTTTGGACT
	Common R: ACTGCAGGGTCCGAGGTATT
β-actin	F: CCTCGCCTTTGCCGATCC
	R: CGCGGCGATATCATCATCC
SNHG3	F: CAGTGGTCGCTTCTTCTCCTT
	R: GCATGAAATGCACCTCAAT
